# Target-prioritized IMRT for nasopharyngeal carcinoma with tumor proximity to the spinal cord: clinical feasibility and long-term outcomes

**DOI:** 10.3389/fonc.2026.1878456

**Published:** 2026-07-06

**Authors:** Yang Li, Wenxuan Huang, Yuanyuan Xu, Han Gao, Lijun Wang, Shengfu Huang, Xia He, Lirong Wu

**Affiliations:** 1 Department of Radiation Oncology, Affiliated Cancer Hospital of Nanjing Medical University & Jiangsu Cancer Hospital & Jiangsu Institute of Cancer Research, Nanjing, China; 2 Department of Radiation Oncology, Changshu Hospital Affiliated to Soochow University, Changshu No. 1 People’s Hospital, Changshu, China

**Keywords:** dosimetry, intensity-modulated radiotherapy, long-term outcomes, nasopharyngeal carcinoma, radiation myelopathy, spinal cord proximity

## Abstract

**Objectives:**

For locally advanced nasopharyngeal carcinoma (NPC) with tumor close to the cervical spinal cord, strict spinal cord constraints may compromise target coverage. We evaluated the feasibility and safety of a target-prioritized IMRT strategy in this setting.

**Materials and methods:**

We retrospectively reviewed 909 consecutive patients with stage III—IVB NPC treated with IMRT between 2012 and 2022. Among 145 patients with tumour-cord proximity, 106 (73.1%) received a target-prioritized strategy, when adequate target coverage could not be achieved under conventional constraints. Dosimetric parameters were extracted from dose-volume histograms. Predictors were evaluated by logistic regression and a cumulative cranio-cervical burden score. Outcomes were analyzed using Kaplan-Meier and log-rank test.

**Results:**

With a median follow-up of 76.11 months, no clinically diagnosed radiation myelopathy (RM) was observed in the overall cohort (upper one-sided 95% CI, 0.33%). The 5-year local relapse–free survival (LRFS) and overall survival (OS) were 92.8% and 86.8%. The cumulative burden score, based on occipital base, occipital condyle, and atlanto-dental interval involvement, was associated with target-prioritized planning (trend OR, 1.906; P = 0.001). In the target-prioritized cohort, median cord Dmax was 56.2 Gy, and 22 patients (20.8%) had a Dmax >60 Gy, while high-dose volumes remained limited. With a median follow-up of 93.24 months in this cohort, 5-year LRFS and OS rates were 87.9% and 74.2%, respectively, and no clinically diagnosed RM was observed (upper one-sided 95% CI, 2.8%).

**Conclusion:**

With strict dose-volume control and image guidance, target-prioritized strategy appears feasible in selected NPC cases but does not support modification of current spinal cord constraints.

## Introduction

Radiotherapy is the cornerstone of treatment for non-metastatic nasopharyngeal carcinoma (NPC), and contemporary intensity-modulated radiotherapy (IMRT) achieves excellent locoregional control (1, 2). However, when the tumor extends posteriorly and lies in close proximity to the cervical spinal cord, achieving adequate target coverage while respecting spinal cord dose constraints becomes a major planning challenge.

Current international guidelines consistently emphasize strict spinal cord protection (3, 4), typically recommending a planning objective of spinal cord planning organ-at-risk volume (PRV) Dmax not exceed 45 Gy and a maximum acceptable limit of 50 Gy, to prevent radiation myelopathy (RM). While these recommendations are effective for the majority of patients, they provide limited guidance for cases in which tumors are in close proximity to the spinal cord and adequate target coverage cannot be achieved within PRV constraints. In clinical practice, such cases are often managed with a “spinal cord–prioritized” strategy, in which target coverage is compromised to preserve spinal cord safety. However, compromising target coverage may lead to underdosage of high-risk targets and potentially suboptimal tumor control, particularly in T3–T4 disease (5). For such patients, achieving an appropriate balance between toxicity and therapeutic efficacy has long been a clinical concern. It is also important to note that many commonly used spinal cord dose constraints were derived from data and practice patterns from the era of conventional radiotherapy, in which the entire spinal cord was irradiated relatively uniformly (6). In contrast, under modern IMRT/IGRT techniques, high-dose regions within the spinal cord typically present as very small-volume, geometrically confined hotspots. Previous studies suggest that the risk of RM is related not only to point dose but also to the volume of spinal cord exposed to high doses (7). This suggests that, under IMRT with strict control of high-dose volumes and IGRT-based setup verification, a target-prioritized strategy may be clinically feasible while preserving therapeutic efficacy through moderate relaxation of spinal cord constraints to maintain adequate target coverage. However, this hypothesis currently lacks supporting evidence from large cohorts. To address this evidence gap, since 2012 our center applied a target-prioritized strategy in selected NPC cases in which conventional spinal cord constraints and adequate target coverage could not be simultaneously achieved. The present study retrospectively evaluates its dosimetric characteristics and long-term clinical outcomes.

## Materials and methods

### Patient selection and imaging

We retrospectively included consecutive patients with stage III–IVB nasopharyngeal carcinoma (NPC) who received definitive IMRT between January 2012 and December 2022. Patients with prior head and neck radiotherapy, incomplete clinical records, or insufficient follow-up data were excluded. A total of 909 patients were eligible for analysis. This study was approved by the Institutional Review Board.

Pretreatment imaging included magnetic resonance imaging of the nasopharynx and neck. Tumour to cord proximity was assessed on fused planning images. The GTV to cervical spinal cord distance was defined as the minimum distance measured on axial fused planning images between the gross tumor volume and the cervical spinal cord contour. Patients with a distance of 2 cm or less were classified as the tumor cord proximity subgroup. The 2 cm criterion served as a prespecified institutional screening flag for closer planning evaluation and did not constitute a direct indication for target-prioritized planning (**Supplementary Table 1**).

### Radiation technique

Patients were placed supine and immobilized with a thermoplastic mask for precision. Inverse IMRT planning was performed using the Varian Inspiration Platform (version 10.0). Target and organ at risk delineation followed institutional protocols and international recommendations. Target volumes included the gross tumor volume (GTV), which comprises the primary tumor (GTVp) and involved lymph nodes (GTVn). Clinical target volume (CTV)1 includes the GTV with 5–10 mm margins, covering the nasopharynx, parapharyngeal space, and retropharyngeal nodal regions. CTV2 encompasses CTV1 plus an additional 3–10 mm margin for regions with diminished microscopic disease risk, positive lymph node involvement, and elective cervical regions per RTOG and EORTC guidelines. Accounting for setup uncertainties, 3mm margins were added to GTVnx, GTVnd, CTV1, and CTV2 to generate PGTVnx, PGTVnd, PTV1, and PTV2. Prescribed doses were PGTVnx 66–74 Gy in 32–34 fractions, PGTVnd 64–70 Gy in 32–34 fractions, PTV1 56–60 Gy in 32 fractions, and PTV2 50.4 Gy in 28 fractions (once daily, 5 fractions per week). In-field hotspots up to 110% were permitted within the PGTV. When imaging confirmed residual disease, an additional boost of 2–4 Gy in 1–2 fractions was delivered. To ensure accurate delivery, all patients underwent image guidance using cone-beam computed tomography (CBCT), which was performed daily for the first three fractions and at least three times per week thereafter to monitor setup accuracy and anatomical changes. Per institutional practice, a repeat planning CT was obtained at approximately fraction 15 to assess anatomical changes. When significant change was identified, an adapted plan was generated for the remaining fractions. Otherwise, the initial plan was used throughout treatment. Organs at risk were evaluated using dose-volume histograms (DVHs) with reference to RTOG 0225 dose constraints.

### Target-prioritized planning strategy

Since 2012, our center has employed a target-prioritized strategy for selected patients with nasopharyngeal carcinoma whose lesions were in close proximity to the cervical spinal cord and in whom target coverage and spinal cord sparing could not be simultaneously achieved during treatment planning. During pre-planning review, cases with a GTV-to-spinal cord distance of 2 cm or less were flagged using this predefined threshold as candidates for closer planning evaluation, rather than as a direct indication for target-prioritized planning. For patients with tumour–cord proximity, treatment planning was initially performed under conventional spinal cord PRV constraints, with a planning objective of PRV Dmax <45 Gy and a maximum acceptable limit of 50 Gy. Each case was first planned by a senior medical physicist. If institutional target coverage goals could not be achieved under conventional PRV constraints, a supervising senior physicist independently reviewed the plan and attempted further cord-sparing optimization. Institutional target coverage goals required D95 ≥100% of the prescription dose for the relevant planning target volumes (PTVs). For the near-minimum dose D98, a three-level compliance criterion was applied: the ideal objective was D98 ≥98% of the prescription dose; the acceptable lower limit was D98 ≥95% of the prescription dose; and, in exceptional circumstances such as when the target was immediately adjacent to critical organs at risk and dose compromise was necessary, D98 was required to remain ≥93% of the prescription dose. Plans with D98 <93% of the prescription dose were considered unacceptable for target coverage. Only when both physicists confirmed that institutional target coverage goals could not be achieved under conventional spinal cord PRV constraints was the case classified as requiring a target-prioritized strategy and escalated to the attending physician. Otherwise, the case was assigned to the non-target-prioritized group because acceptable target coverage remained achievable within conventional spinal cord PRV constraints. In our institutional practice, a target-prioritized strategy referred to a planning approach in which adequate coverage of the GTV and CTV was prioritized when conventional spinal cord PRV constraints and adequate coverage of these target volumes could not be simultaneously achieved. This did not represent uncontrolled dose escalation to the spinal cord. Rather, spinal cord PRV constraints were selectively relaxed only when necessary on a case-by-case basis, with strict effort to minimize the volume and longitudinal extent of any hotspot. In practice, the need for this strategy was more often driven not by direct abutment of the GTV to the spinal cord, but by close proximity or overlap between the spinal cord PRV and CTV1. The attending physician informed the patient of the necessity, rationale, potential benefits, and potential risks of selective PRV relaxation, and written informed consent was obtained before selective PRV relaxation was authorized. During treatment delivery, strict image guidance and setup verification were maintained to reduce geometric uncertainty.

### Spinal cord dosimetry

In clinical planning, spinal cord dose constraints were applied to the spinal cord planning risk volume (PRV), defined as the true spinal cord with a uniform 3 mm margin according to institutional practice. Dosimetric parameters were extracted from cumulative Plan-Sum dose-volume histograms (DVHs). For patients who underwent adaptive replanning, Plan-Sum DVHs represented the composite dose from the initial plan and any adapted plan. Parameters were reported for both the true spinal cord contour and the PRV, and included Dmax, Dmean, D0.03cc, D0.1cc, D1cc, and the absolute volumes receiving 45, 50, 55, and 60 Gy or more (V45–V60, cm³). The craniocaudal longitudinal extent (mm) of cord receiving ≥45, 50, 55, and 60 Gy was also measured.

### Follow-up

Upon IMRT completion, patients were followed up every 3 months during the initial 2 years, every 6 months for the succeeding 3 years, and annually thereafter. At each follow-up visit, a neurological examination was performed by the attending physician, and a head and neck MRI was obtained to monitor local control and to detect any radiologic signs of RM. Radiation myelopathy was defined as new myelopathic symptoms (motor weakness, sensory deficit, or autonomic dysfunction) at or below the irradiated cord level, with corroborating MRI signal changes, after exclusion of disease recurrence or cord compression.

### Statistical methods

Continuous variables were compared using the independent-samples t test, while a chi-square or Fisher’s exact test was employed to compare categorical variables. The Kaplan–Meier method was used to estimate the time-to-event outcomes, including overall survival (OS) and local relapse-free survival (LRFS). Between-group differences were evaluated using the log-rank test. To identify anatomic predictors of adopting a target-prioritized strategy, candidate anatomic structures adjacent to the cervical spinal cord were evaluated using univariable logistic regression, and variables with P < 0.10 were entered into a multivariable logistic regression model. To address potential collinearity among anatomically adjacent structures, a cumulative cranio-cervical burden score was constructed from factors reaching P ≤ 0.05 in univariable analysis; trend was assessed by entering the score as a continuous predictor. Follow-up duration was estimated using the reverse Kaplan-Meier method. Results are reported as odds ratios with 95% confidence intervals. A *p* < 0.05 was considered statistically significant. R Statistical Software (version 4.2.2; The R Foundation for Statistical Computing, Vienna, Austria), and the Free Statistics analysis platform (version 2.1; Beijing, China) were utilized for all analyses and visualizations.

## Results

### Baseline characteristics and treatment outcomes

**Figure 1:**
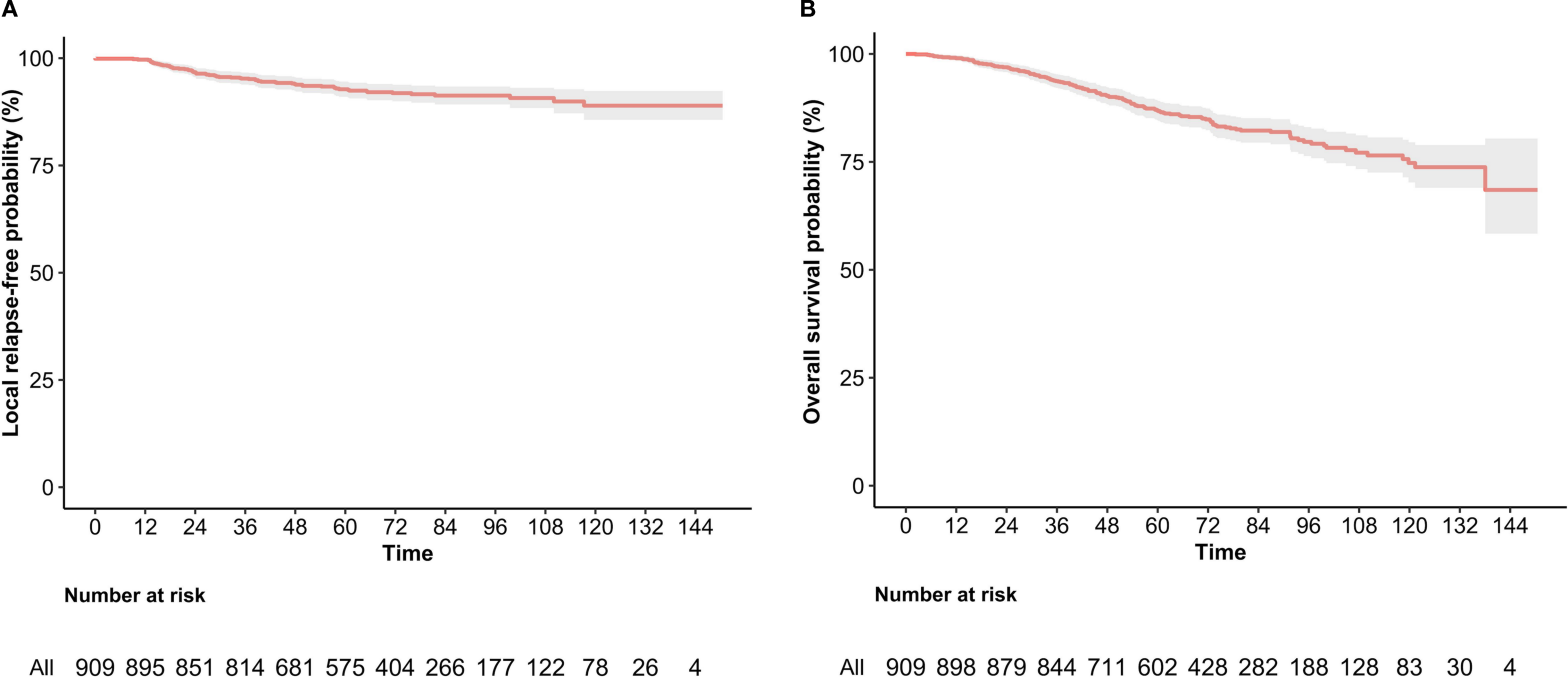
Kaplan–Meier estimates of local relapse-free survival and overall survival in the overall cohort. Kaplan–Meier curves showing local relapse-free survival **(A)** and overall survival **(B)** in the overall cohort of 909 patients with locoregionally advanced nasopharyngeal carcinoma treated with definitive IMRT. Shaded areas indicate 95% confidence intervals. Numbers at risk are shown below each panel.

A total of 909 patients with locoregionally advanced NPC who were treated with IMRT at the Jiangsu Cancer Hospital between 2012 and 2022 were included in this study. Baseline characteristics are summarized in **Table 1**. With a median follow-up of 76.11 months (IQR: 61.6–97.5 months), no clinically diagnosed RM was observed in the entire cohort (upper one-sided 95% CI 0.33%). Treatment outcomes were favorable, with 5-year local relapse–free survival (LRFS) and overall survival (OS) rates of 92.8% and 86.8%, respectively (**Figure 1**).

**Table 1:** Baseline characteristics and outcomes of the overall cohort (n = 909).

Variables	Total (n = 909)
Age, Mean ± SD	49.6 ± 13.2
Sex, n (%)	
Female	248 (27.3%)
Male	661 (72.7%)
T stage, n (%)	
1	104 (11.4%)
2	90 (9.9%)
3	319 (35.1%)
4	396 (43.6%)
N stage, n (%)	
0	50 (5.5%)
1	380 (41.8%)
2	341 (37.5%)
3	138 (15.2%)
Clinical stage, n (%)	
III	414 (45.5%)
IV	495 (54.5%)
Median follow-up, months	70.6 months (IQR 51.8–90.3)
5-year local control	92.80%
5-year overall survival	86.80%
GTV-cord distance, n (%)	
≤2cm	145(16.0%)
>2cm	764 (84.0%)
Radiation myelopathy, n	0

Among the 909 patients, 145 cases (16.0%) presented with tumors in close proximity to the cervical spinal cord (GTV–cord distance ≤ 2 cm; **Table 2**). Among them, 106 (73.1%) required a target-prioritized IMRT strategy, whereas 39 (26.9%) did not. The 39 patients in the non-target-prioritized group did not require this strategy because acceptable target coverage remained achievable under conventional spinal cord PRV constraints during formal planning review. Compared with the non–target-prioritized group, patients in the target-prioritized group received a slightly lower dose per fraction. Apart from this, the two groups were comparable in baseline characteristics, including age, sex, T stage, N stage, and overall clinical stage, with no statistically significant differences observed (*p* > 0.05). Regarding systemic therapy in the target-prioritized cohort, 57 (53.8%) received induction chemotherapy (primarily TPF or cisplatin/taxane regimens), 74 (69.8%) received concurrent platinum-based chemotherapy, and 18 (17.0%) received adjuvant chemotherapy. Given that nearly three-quarters of patients in the GTV–cord ≤ 2 cm subgroup required a target-prioritized plan, we next investigated which anatomical extensions were associated with adopting this strategy.

**Table 2:** Baseline patient and treatment characteristics (*N* = 145).

Variable	Total (n = 145)	Non-target-prioritized group (n = 39)	Target-prioritized group (n = 106)	p
Age, years				0.356
Mean ± SD	47.9 ± 14.4	46.0 ± 14.9	48.5 ± 14.3	
Gender				0.859
Female	32 (22.1%)	9 (23.1%)	23 (21.7%)	
Male	113 (77.9%)	30 (76.9%)	83 (78.3%)	
T stage				0.855
3	16 (11.0%)	4 (10.3%)	12 (11.3%)	
4	129 (89.0%)	35 (89.7%)	94 (88.7%)	
N stage				0.74
0	6 (4.1%)	2 (5.1%)	4 (3.8%)	
1	80 (55.2%)	19 (48.7%)	61 (57.5%)	
2	41 (28.3%)	13 (33.3%)	28 (26.4%)	
3	18 (12.4%)	5 (12.8%)	13 (12.3%)	
Clinical Stage				0.88
III	14(9.7%)	4(10.3%)	10(9.4%)	
IV	131(90.3%)	35(89.7%)	96(90.6%)	
Induction Chemotherapy				0.66
No	70 (48.3%)	20 (51.3%)	50 (47.2%)	
Yes	75 (51.7%)	19 (48.7%)	56 (52.8%)	
Concurrent chemotherapy				0.076
No	42 (29.0%)	7 (17.9%)	35 (33%)	
Yes	103 (71.0%)	32 (82.1%)	71 (67%)	
Adjuvant chemotherapy				0.951
No	126 (86.9%)	34 (87.2%)	92 (86.8%)	
Yes	19 (13.1%)	5 (12.8%)	14 (13.2%)	
GTV Dose (Gy)				0.091
Mean ± SD	68.81 ± 1.8	69.23 ± 1.6	68.66 ± 1.9	
Dose per Fraction (Gy)				0.005
Mean ± SD	2.12 ± 0.06	2.14 ± 0.06	2.11 ± 0.06	

### Anatomical predictors for target‐prioritized planning

**Figure 2:**
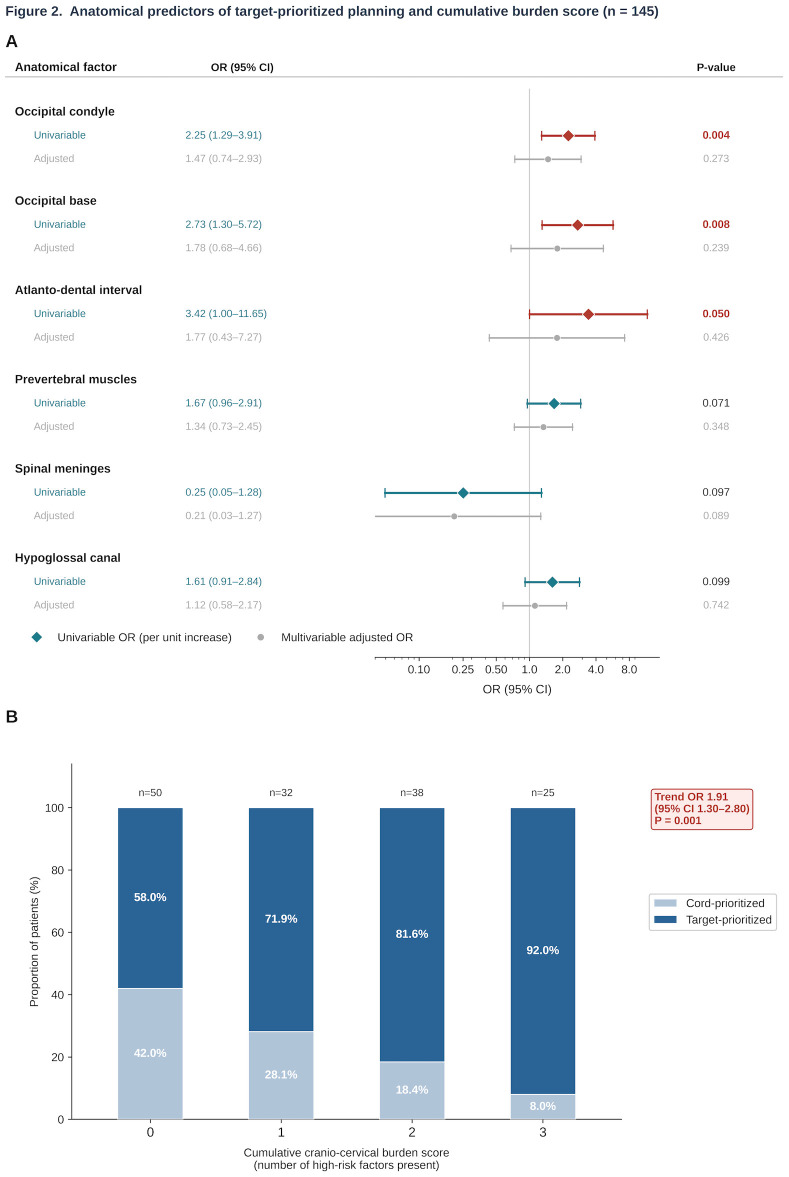
Anatomical predictors of a target-prioritized strategy and cumulative cranio-cervical burden score. **(A)** Forest plot of the six candidate anatomical variables identified by univariable screening at P <0.10. For each factor, the upper row (diamond) shows the univariable OR, and the lower row (circle) shows the multivariable adjusted OR when all six candidates were entered simultaneously into the model (EPV = 17.7). ORs were estimated per one-level increase for ordinal 0/1/2 variables or for presence versus absence for binary variables. **(B)** Proportion of patients requiring target-prioritized strategy according to the cumulative cranio-cervical burden score. The score was calculated as the sum of three dichotomized factors: occipital base, occipital condyle, and atlanto-dental interval involvement. The proportion requiring target-prioritized strategy increased from 58.0% (29/50) for score 0, to 71.9% (23/32) for score 1, 81.6% (31/38) for score 2, and 92.0% (23/25) for score 3. The trend OR was 1.906 per additional factor (95% CI 1.298–2.799; P = 0.001).

To identify anatomical determinants of adopting a target-prioritized strategy, we evaluated 25 anatomical structures potentially adjacent to the cervical spinal cord (**Supplementary Table 2**). Univariable logistic regression was used to assess the association between each structure and the use of a target-prioritized strategy. Variables with *P* < 0.1 in univariable analysis were entered into a multivariable logistic regression model. Three structures reached P ≤0.05 in univariable analysis: occipital condyle involvement (OR 2.248, 95% CI 1.29–3.91; P = 0.004), occipital base involvement (OR 2.728, 95% CI 1.30–5.72; P = 0.008), and atlanto-dental interval involvement (OR 3.416, 95% CI 1.00–11.65; P = 0.050). Three additional structures showed borderline associations (P ≤0.10): prevertebral muscles (P = 0.071), spinal meninges (P = 0.097), and hypoglossal canal (P = 0.099). When all six candidate variables were entered simultaneously into a multivariable logistic regression model, all factors were attenuated after mutual adjustment, consistent with collinearity among these anatomically adjacent posterior cranio-cervical structures (EPV = 106/6 = 17.7; McFadden R² = 0.098; **Figure 2A**). No single structure remained independently significant. Therefore, we constructed a cumulative cranio-cervical burden score based on the three factors with P ≤0.05 in univariable analysis, namely occipital base, occipital condyle, and atlanto-dental interval involvement. Each factor was dichotomized as absent (0) or present (>0), and the score was calculated as the sum of these factors, ranging from 0 to 3. The proportion requiring target-prioritized planning increased monotonically with increasing burden score (**Figure 2B**). The trend OR was 1.906 per additional factor (95% CI 1.298–2.799; P = 0.001). Patients with all three factors present had an OR of 8.328 (95% CI 1.767–39.244; P = 0.007) compared with patients with no factors (**Figure 2B**; **Supplementary Table 2**).

### Spinal cord and PRV dosimetry in the target-prioritized cohort

**Figure 3:**
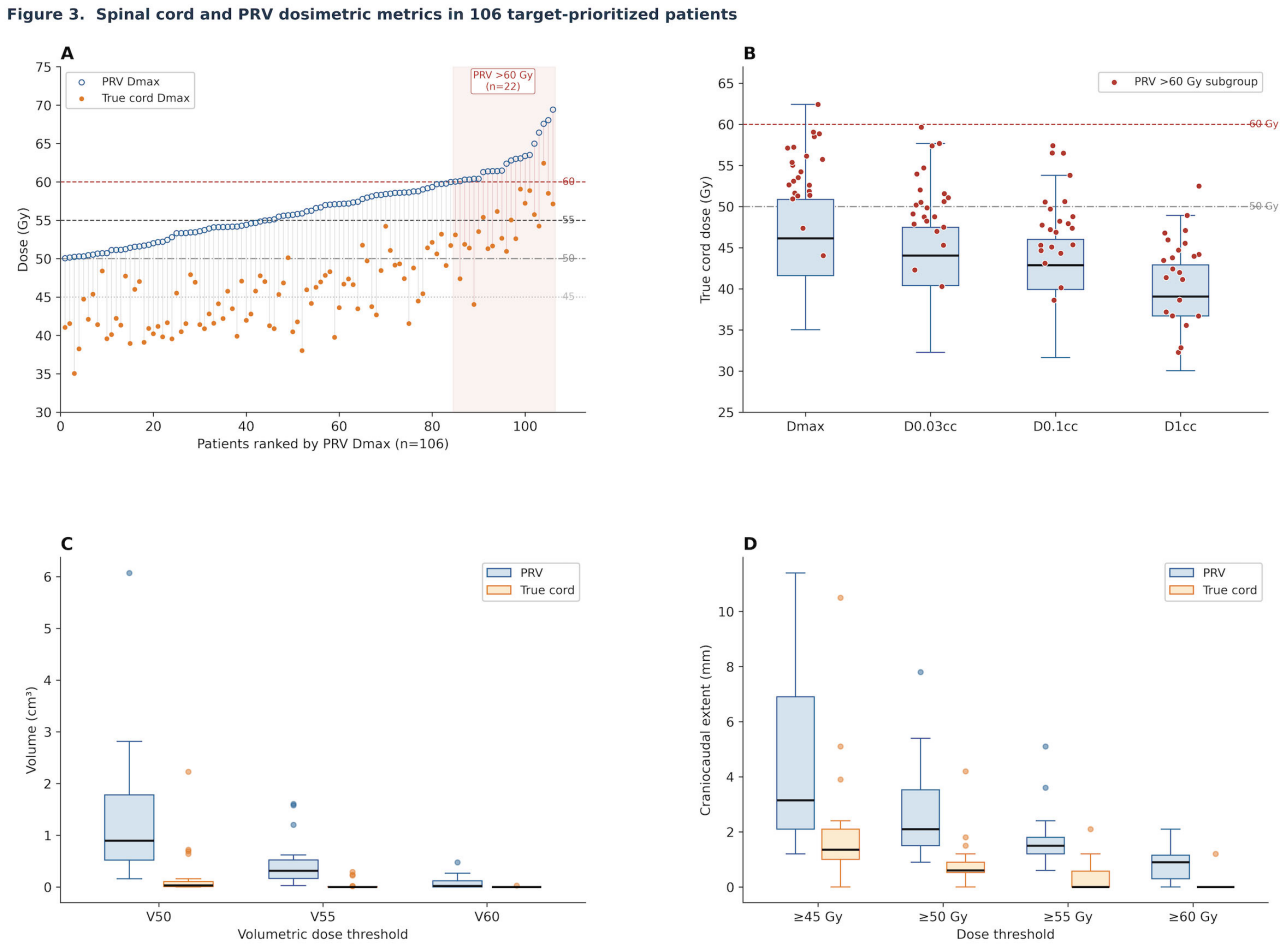
Spinal cord dosimetry in the target-prioritized cohort. **(A)** Paired Dmax values for the 3-mm spinal cord PRV and the true spinal cord, with patients ranked in ascending order of PRV Dmax. Vertical lines connect values within each patient. The shaded region indicates the PRV Dmax >60 Gy subgroup (n = 22). Horizontal reference lines are shown at 45, 50, 55, and 60 Gy. **(B)** Maximum and near-maximum true spinal cord dose metrics across the target-prioritized cohort, including Dmax, D0.03cc, D0.1cc, and D1cc. Boxes show the median and interquartile range, and whiskers show the 5th to 95th percentiles. Red dots indicate the 22 patients in the PRV Dmax >60 Gy subgroup overlaid on the cohort distribution. **(C)** High-dose volumes (V50, V55, and V60) of the 3-mm spinal cord PRV and the true spinal cord within the PRV Dmax >60 Gy subgroup (n = 22). Boxes show the median and interquartile range, whiskers extend to 1.5 times the interquartile range, and individual outliers are shown as dots. **(D)** Craniocaudal extent of regions receiving at least 45, 50, 55, and 60 Gy for the 3-mm spinal cord PRV and the true spinal cord within the same subgroup.

In the target-prioritized cohort (n = 106), PRV and true spinal cord dosimetric parameters are summarized in **Table 3** and illustrated in **Figure 3**. The median PRV Dmax was 56.2 Gy (IQR 53.4–59.3 Gy), and 22 patients (20.8%) had a PRV Dmax > 60 Gy. The median PRV D0.03cc and D1cc were 52.2 Gy (IQR, 49.6–56.5 Gy) and 44.0 Gy (IQR 41.7–47.1Gy), respectively. In the Dmax > 60 Gy subgroup (n = 22), the median Dmax was 63.1 Gy (IQR 61.2–64.9 Gy). High-dose volumes remained limited. The median PRV V50, V55, and V60 were 0. 897 cm³ (IQR 0.522–1.779 cm³), 0.315 cm³ (IQR 0.206–0.541 cm³), and 0.020 cm³ (IQR 0.001–0.124 cm³), respectively.

**Table 3:** Spinal cord and PRV dosimetric parameters in the target-prioritized cohort.

Parameter	Full cohort (n = 106)		PRV Dmax > 60 Gy subgroup (n = 22)	
	PRV cord	True cord	PRV cord	True cord
Point and near-maximum doses (Gy)				
Dmax	56.2 (53.4–59.3)	46.1 (41.6–50.9)	61.9 (60.6–63.5)	53.9 (51.7–56.9)
Dmean	31.1 (28.0–34.9)	31.3 (28.2–35.1)	30.2 (28.0–34.5)	30.3 (28.7–34.0)
D0.03cc	52.2 (49.6–56.5)	44.0 (40.4–47.5)	59.6 (58.4–61.8)	50.0 (48.0–51.9)
D0.1cc	50.4 (47.0–54.7)	42.9 (39.9–46.0)	57.9 (56.6–60.1)	47.6 (45.1–50.3)
D1cc	44.0 (41.7–47.1)	39.1 (36.7–42.9)	49.4 (46.3–52.5)	42.9 (37.5–45.3)
High-dose volumes (cm³)				
V40	3.245 (1.897–11.522)	0.561 (0.083–3.503)	7.312 (3.006–13.895)	1.899 (0.548–5.030)
V45	0.764 (0.210–2.168)	0.005 (0.000–0.287)	2.710 (1.298–4.411)	0.315 (0.119–0.977)
V50	0.131 (0.023–0.462)	0.000 (0.000–0.001)	0.897 (0.518–1.781)	0.031 (0.005–0.105)
V55	0.004 (0.000–0.085)	0.000 (0.000–0.000)	0.315 (0.163–0.520)	0.000 (0.000–0.005)
V60	0.000 (0.000–0.000)	0.000 (0.000–0.000)	0.020 (0.001–0.117)	0.000 (0.000–0.000)
Craniocaudal hotspot extent (mm)				
≥45 Gy	2.5 (1.5–4.5)	0.6 (0.0–1.5)	3.1 (2.1–6.9)	1.4 (1.0–2.1)
≥50 Gy	1.2 (0.9–2.1)	0.0 (0.0–0.3)	2.1 (1.5–3.5)	0.6 (0.5–0.9)
≥55 Gy	0.6 (0.0–1.2)	0.0 (0.0–0.0)	1.5 (1.2–1.8)	0.0 (0.0–0.6)
≥60 Gy	0.0 (0.0–0.0)	0.0 (0.0–0.0)	0.9 (0.3–1.1)	0.0 (0.0–0.0)
True cord Dmax exceeding clinical thresholds, n (%)				
> 45 Gy		61/106 (57.5%)		21/22 (95.5%)
> 50 Gy		29/106 (27.4%)		20/22 (90.9%)
> 60 Gy		1/106 (0.9%)		1/22 (4.5%)

The median true spinal cord Dmax was 46.1 Gy (IQR, 41.6–50.9 Gy), with 29 patients (27.4%) exceeding 50 Gy and 1 patient (0.9%) exceeding 60 Gy. The median true spinal cord D0.03cc was 44.0 Gy (IQR, 40.4–47.5 Gy), D0.1cc was 42.9 Gy (IQR, 39.9–46.0 Gy), and D1cc was 39.1 Gy (IQR, 36.7–42.9 Gy). The median true spinal cord V45 and V50 were 0.005 cm³ (IQR, 0.000–0.287 cm³) and 0.000 cm³ (IQR, 0.000–0.001 cm³), respectively. The median craniocaudal extent of true spinal cord receiving ≥45 Gy was 0.6 mm (IQR, 0.0–1.5 mm), and that receiving ≥50 Gy was 0.0 mm (IQR, 0.0–0.3 mm). Among the 22 patients with PRV Dmax >60 Gy, the median true spinal cord Dmax was 53.9 Gy (IQR, 51.7–56.9 Gy), and 1 patient had a true spinal cord Dmax >60 Gy.

### Target coverage and long-term outcomes in the target-prioritized cohort

Achieved target coverage for GTV1 and CTV1 in the 106 target-prioritized patients is summarized in **Table 4**. D95 was at least the prescription dose in all 106 patients for both GTV1 and CTV1. For the D98 compliance criterion, 106/106 patients (100%) achieved D98 ≥95% of the prescription dose for GTV1, and 103/106 patients (97.2%) achieved D98 ≥95% of the prescription dose for CTV1. All patients achieved D98 ≥93% of the prescription dose for both GTV1 and CTV1, indicating that no unacceptable target undercoverage occurred in the target-prioritized cohort.

**Table 4:** Achieved target coverage in the target-prioritized cohort.

Target volume	D95 ≥ Dp	D98 ≥98% Dp	D98 ≥95% Dp	D98 ≥93% Dp
GTV1	106/106 (100%)	98/106 (92.5%)	106/106 (100%)	106/106 (100%)
CTV1	106/106 (100%)	91/106 (85.8%)	103/106 (97.2%)	106/106 (100%)

Dp, prescription dose; D95, dose received by 95% of the target volume; D98, dose received by 98% of the target volume.

**Figure 4:**
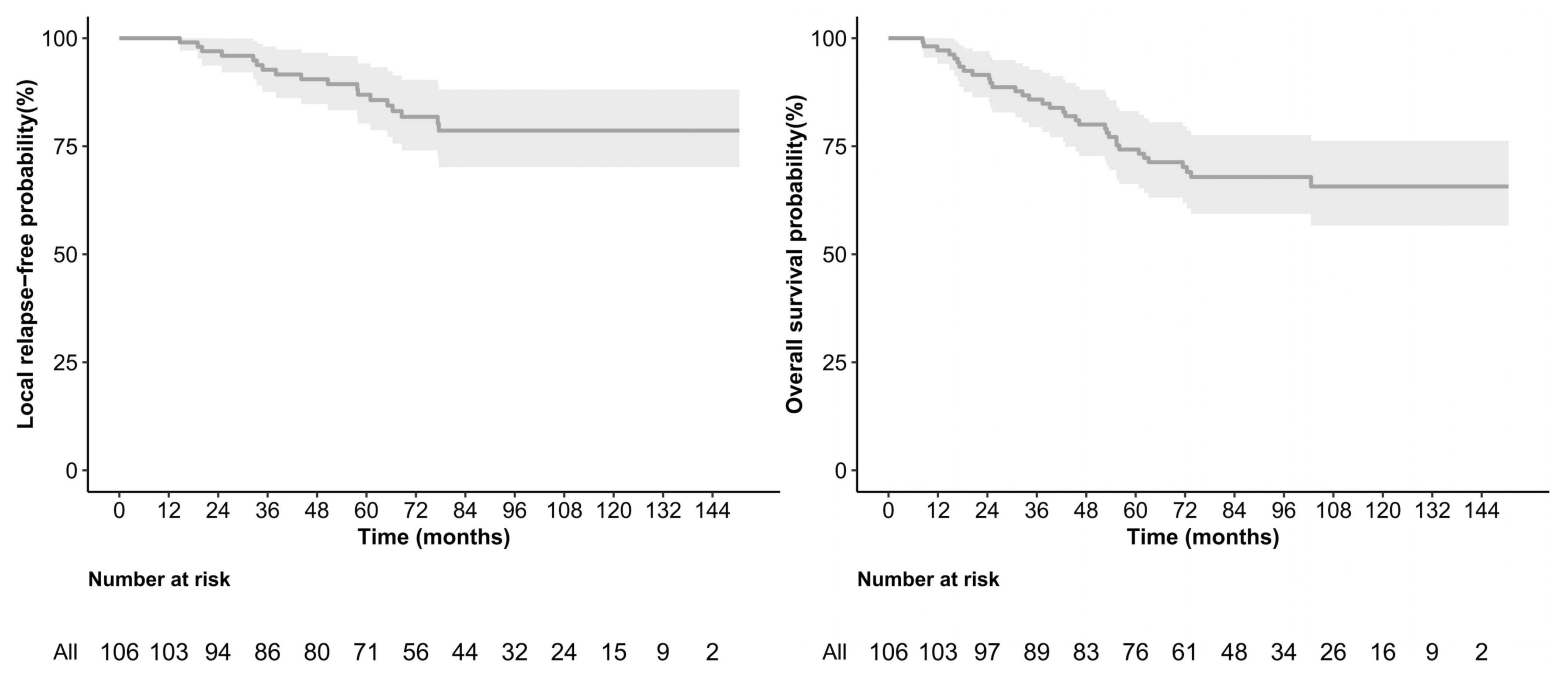
Kaplan–Meier estimates of long-term outcomes in the target-prioritized cohort. Kaplan–Meier curves showing local relapse-free survival and overall survival in the 106 patients managed with a target-prioritized strategy. Shaded areas indicate 95% confidence intervals. Numbers at risk are shown below each panel.

As of December 2024, the median follow-up duration for the 106 patients managed with a target-prioritized strategy was 93.24 months (range 7.4–150.5), and 78 of 106 (73.6%) had at least 5 years of follow-up. The 5-year LRFS, OS, and distant metastasis-free survival (DMFS) rates were 87.9%, 74.2%, and 71.6%, respectively (**Figure 4**). All local recurrences were intra-field failures, and distant metastasis was the predominant pattern of failure. During follow-up, no local failure events were accompanied by neurologic symptoms attributable to the spinal cord or brainstem. Among the 78 patients with available documentation of spinal cord-related symptoms, five (6.4%) reported transient Lhermitte’s sign, and all cases resolved spontaneously. None of these patients met the diagnostic criteria for RM. In the entire cohort of 106 patients, no clinically diagnosed RM was observed (upper one-sided 95% CI, 2.8%). In a sensitivity analysis stratified by treatment era, 59/106 (55.7%) of target-prioritized patients were treated in 2012–2016 and 47/106 (44.3%) in 2017–2022; 5-year LRFS was 87.7% vs 84.5% respectively (log-rank P = 0.450; **Supplementary Figure S1**).

## Discussion

This study demonstrates that for locoregionally advanced NPC with primary tumors in close proximity to the cervical spinal cord, a target-prioritized IMRT strategy is a clinically feasible solution to the conflict between target coverage and organ-at-risk protection. In our cohort of 909 patients with stage III-IVB, long-term tumor control and survival outcomes were generally favorable compared with previous reports (8), and no RM was observed during follow-up. Meanwhile, 16.0% of patients in our cohort had a GTV-to-cord distance of 2 cm or less. This suggests that while RM is uncommon in the IMRT era, the anatomical situation of tumour-cord proximity is not rare in locoregionally advanced disease. Notably, nearly three-quarters of these cases ultimately required a target-prioritized strategy, prioritizing target coverage by intentionally exceeding spinal cord dose constraints. Despite this, long-term tumor control remained robust, and no cases of RM were observed. These findings suggest that in this specific clinical setting, a target-prioritized strategy is a viable option.

We identified specific anatomical patterns associated with the need for a target-prioritized strategy. Three posterior craniocervical structures, namely the occipital condyle, occipital base, and atlantodental interval, were each associated with target-prioritized planning in the univariable analysis. However, these associations were attenuated after mutual adjustment in the multivariable analysis, consistent with collinearity among these anatomically adjacent structures. Rather than reporting potentially spurious independent effects, we constructed a cumulative burden score to integrate this shared anatomical information. The score demonstrated a clear dose-response relationship: patients with all three factors present had a 92.0% probability of requiring target-prioritized planning (OR, 8.328 compared with patients with no factors; P = 0.007). These findings suggest that the cumulative burden of posterior craniocervical junction involvement, rather than any single structure, drives the likelihood of cord-target conflict. The score may serve as a clinically interpretable exploratory indicator for pre-planning risk assessment to identify patients in whom target coverage is most likely to require PRV constraint relaxation. For patients with a high cumulative anatomical burden involving the cranio-cervical junction, particularly those with concurrent involvement of the occipital condyle, occipital base, and atlantodental interval, the elective high-risk clinical target volume (CTV1) is more likely to overlap with the spinal cord PRV. In such cases, meticulous contouring of the spinal cord and a more prudent definition of CTV1 boundaries are warranted to mitigate risk.

In current head and neck radiotherapy practice, spinal cord constraints are often treated as hard limits, with Dmax 45 Gy as the planning objective and relaxation to 50 Gy when target coverage is compromised (3, 9–13). In the present study, the spinal cord PRV served as the clinical structure for plan evaluation and constraint assessment, whereas true cord dosimetry was analyzed separately to characterize actual cord exposure. In the target-prioritized cohort, the median PRV Dmax was 56.2 Gy and PRV D0.03cc was 52.2 Gy, with PRV Dmax exceeding 60 Gy in 22 patients and 65Gy in 2 patients, substantially exceeding the conventional PRV-based thresholds, yet no clinically diagnosed RM was observed during a median follow-up of 93.24 months (upper 95% CI 2.8%, 0/106). Further evaluation of true cord doses in the target-prioritized cohort showed a median true cord Dmax of 46.1 Gy and a median true cord D0.03cc of 44.0 Gy, indicating that the PRV-based metrics reported above overestimated the actual dose delivered to the true cord. Nonetheless, in the PRV Dmax > 60 Gy subgroup, the median true cord D0.03cc reached 50.0 Gy (IQR 48.0–51.9) and D0.1cc reached 47.6 Gy (IQR 45.1–50.3), both clearly exceeding the conventional 45 Gy threshold for the true cord. Despite these elevated doses in both PRV and true cord, the absence of toxicity may be attributable to the extremely limited volume of exposure. Even in the subgroup with PRV Dmax > 60 Gy, the absolute volumes of PRV V55 and V60 remained negligible, confirming that the high-dose exposure manifested as focal hotspots rather than extensive segmental irradiation. True cord dosimetric data further supported this observation. In this subgroup, the median true cord V50 was only 0.031 cm³, and the median craniocaudal extent of true cord receiving ≥50 Gy was only 0.6 mm, indicating that even when true cord doses exceeded conventional thresholds, the spatial extent of exposure remained minimal. A previous study (14) has demonstrated that the risk of RM is closely correlated with the volume of the spinal cord receiving high doses. Moreover, current spinal cord dose constraints are largely derived from historical dose-response data and treatment paradigms from the conventional radiotherapy and early 3D-CRT eras. In the two-dimensional radiotherapy era for nasopharyngeal carcinoma (NPC), treatment was typically delivered using lateral opposed fields, followed by off-cord reduced fields or posterior cervical electron boosts. These plans were based on two-dimensional simulation radiographs without modern volumetric image guidance. Consequently, the spatial relationship between field borders and the cervical spinal cord was often uncertain, and the actual spinal cord dose could differ substantially from the estimated or prescribed dose. This historical context may partly explain why radiation myelopathy was more frequently reported during the conventional radiotherapy era yet has become exceedingly rare in the intensity-modulated radiotherapy (IMRT) era, even in the presence of occasional focal hotspots. In contrast, contemporary IMRT combined with cone-beam CT (CBCT) image guidance enables highly precise contouring and localization, thereby restricting high-dose exposure to small, geometrically confined volumes of the spinal cord. Our data suggest that, when implementing a target-prioritized strategy, localized spinal cord hotspots may be clinically tolerable. However, given the rarity of RM events and limited evidence in the IMRT era, this study provides a real-world reference with detailed dosimetric distributions and long-term follow-up outcomes that may inform future risk assessment and serve as a reference dataset for external validation but is insufficient on its own to support relaxation of current constraints.

Adequate target coverage remains essential for local control. In the present study, patients managed with a target-prioritized strategy represented a clinically challenging subgroup characterized by more extensive disease and greater planning complexity. Achieved target coverage metrics showed that institutional coverage goals for both GTV1 and CTV1 were preserved in the final approved plans, with no unacceptable target undercoverage. Despite this complexity, the 5-year LRFS and OS in this cohort remained acceptable. In particular, the 5-year LRFS of 87.9% appeared to fall within the range of previously reported outcomes for patients with T4 (15) or stage IV (16) nasopharyngeal carcinoma. All local recurrences were intra-field failures, suggesting that failure was more likely related to intrinsic tumour burden or radioresistance than to marginal miss. Taken together, these findings suggest that, in selected patients in whom conventional cord-prioritized planning would compromise target coverage, prudent implementation of a target-prioritized strategy may help maintain satisfactory long-term oncologic outcomes. However, because optimization plans generated before PRV constraint relaxation were not routinely archived, a formal before-and-after comparison could not be performed. Thus, these data reflect the target coverage achieved in the final approved plans but do not quantify the magnitude of improvement attributable to PRV constraint relaxation. The findings should be interpreted as supporting the clinical feasibility of this strategy in selected high-complexity cases, rather than as evidence of superiority over conventional planning approaches or as justification for modifying current spinal cord dose constraints.

The longer follow-up in the target-prioritized cohort (median 93.24 months) reflects earlier accrual rather than a survivorship artifact. Among the 106 patients, 59 (55.7%) were treated in 2012–2016 and 47 (44.3%) in 2017–2022. Era-stratified analysis confirmed that 5-year LRFS did not differ significantly between eras (87.7% vs 84.5%, log-rank P = 0.450), indicating that local control outcomes were not materially affected by changes in planning or image-guidance practice over the study period. In contrast, the earlier treatment era was characterized by less intensive and less effective systemic therapy regimens relative to contemporary standards, which may partly explain both the higher proportion of target-prioritized cases accrued during that period and the relatively lower overall survival observed in the cohort as a whole.

This is the first study to systematically compare true cord and PRV dosimetry in the context of the radiotherapy decision-making dilemma in locoregionally advanced nasopharyngeal carcinoma with a primary tumour proximity to the cervical spinal cord, providing clinical evidence for a target-prioritized strategy in the real-world trade-off between target coverage and spinal cord sparing, yet limitations remain. Since RM is a rare event, the statistical power of this study is insufficient to derive specific risk thresholds for Dmax or small-volume exposure, and the study was not intended to propose new dose-constraint recommendations. As existing head and neck guidelines and planning protocols are primarily framed in terms of physical dose thresholds, we reported spinal cord-related physical dose parameters rather than EQD2-based metrics in the main text. EQD2 conversions (α/β = 2 Gy) are provided in **Supplementary Table 3** for cross-study comparison. Five patients (6.4%) reported transient Lhermitte sign, which resolved spontaneously in all cases; none met the diagnostic criteria for RM. Neurological assessments were documented for 78 of 106 patients (73.6%), and the retrospective nature of symptom ascertainment may have underestimated subclinical neurologic events. Pre-relaxation planning records were not retained per institutional practice, precluding a before-and-after comparison of target coverage. Future multicenter cohorts with prospective and standardized neurologic assessment are needed to validate these findings and improve generalizability.

In summary, for patients with locoregionally advanced NPC in whom the primary tumour in close proximity to the cervical spinal cord, a target-prioritized strategy is a feasible option in clinical practice, with strict setup, planning quality controls, and minimized high-dose volumes. True cord doses were substantially lower than PRV metrics indicated, though true cord D0.03cc in the highest-risk subgroup exceeded the conventional 45 Gy threshold, underscoring the importance of distinguishing between PRV and true cord when interpreting spinal cord dosimetry. A cumulative cranio-cervical burden score based on occipital base, occipital condyle, and atlanto-dental interval involvement may help identify patients most likely to require this strategy and inform pre-planning decision-making and follow-up surveillance. These findings provide real-world dosimetric reference data and are not intended to support modification of current spinal cord dose-constraint recommendations.

## Data Availability

The raw data supporting the conclusions of this article will be made available by the authors, without undue reservation.
