# Supplementary figures and images for: Target-prioritized IMRT for nasopharyngeal carcinoma with tumor proximity to the spinal cord: clinical feasibility and long-term outcomes
Source: Front Oncol. 2026 Jul 6;16:1878456. doi: 10.3389/fonc.2026.1878456 (PMC13381646; doi:10.3389/fonc.2026.1878456)

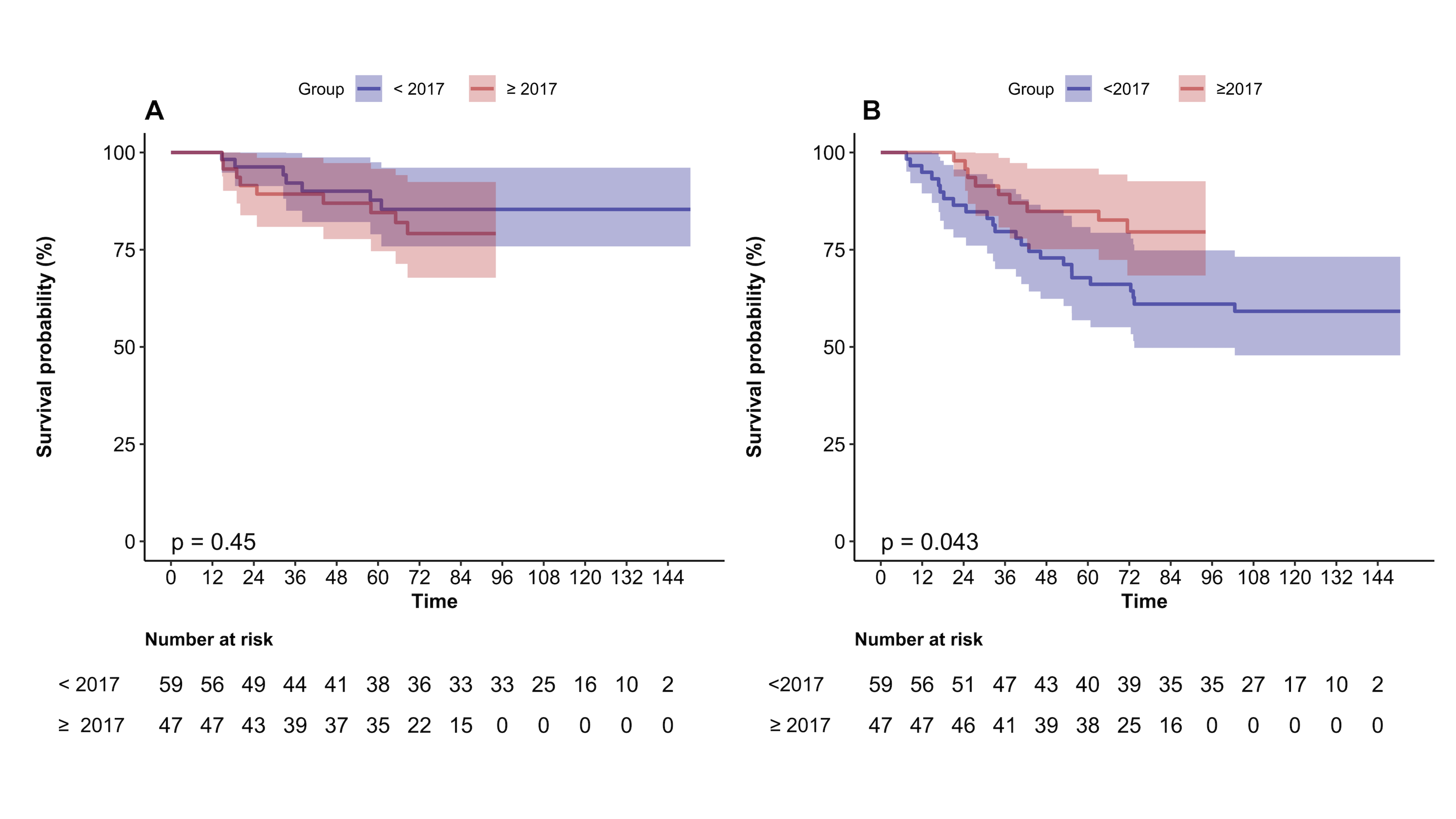

Supplement: Supplementary file 1 [file Image1.tif]
