# Supplementary material for: Target-prioritized IMRT for nasopharyngeal carcinoma with tumor proximity to the spinal cord: clinical feasibility and long-term outcomes
Source: Front Oncol. 2026 Jul 6;16:1878456. doi: 10.3389/fonc.2026.1878456 (PMC13381646; doi:10.3389/fonc.2026.1878456)
Supplement: Supplementary file 2 [file Table1.docx]

**Supplementary Table S1. Sensitivity analysis of the cumulative cranio-cervical burden score across alternative GTV-spinal cord distance thresholds**

The cumulative burden score comprised three dichotomised factors: occipital base involvement, occipital condyle involvement, and atlanto-dental interval involvement. The proximity cohort was defined by GTV-spinal cord distance less than or equal to the stated threshold. The outcome was target-prioritized planning, defined as final cumulative PRV Dmax > 50 Gy.

**Part A. Distribution of target-prioritized planning rates by cumulative burden score**

| Score | <=1.0 cm (N = 43) | <=1.5 cm (N = 109) | <=2.0 cm (main analysis, N = 145) |
| --- | --- | --- | --- |
| 0 factors | 9 / 9 (100.0%) | 37 / 24 (64.9%) | 50 / 29 (58.0%) |
| 1 factor | 10 / 9 (90.0%) | 22 / 19 (86.4%) | 32 / 23 (71.9%) |
| 2 factors | 13 / 12 (92.3%) | 29 / 26 (89.7%) | 38 / 31 (81.6%) |
| 3 factors | 11 / 11 (100.0%) | 21 / 21 (100.0%) | 25 / 23 (92.0%) |

*n shown as total / target-prioritized count, with the percentage representing the proportion of target-prioritized cases within each score category.*

**Part B. Logistic regression results**

| Distance threshold | Cohort N | TP n (%) | Trend OR per additional factor (95% CI) | P | High-burden contrast | High-burden OR (95% CI) | P |
| --- | --- | --- | --- | --- | --- | --- | --- |
| <=1.0 cm | 43 | 41 (95.3%) | Not reported due to extreme outcome imbalance  (TP = 41, SP = 2) | — | Not reported | — | — |
| <=1.5 cm | 109 | 90 (82.6%) | 2.761 (1.484–5.138) | 0.001 | >=2 vs 0 factors | 8.486 (2.204–32.678) | 0.002 |
| <=2.0cm (main analysis) | 145 | 106 (73.1%) | 1.906 (1.298–2.799) | 0.001 | >=2 vs 0 factors | 4.345 (1.763–10.707) | 0.001 |

CI = confidence interval. OR = odds ratio. TP = target-prioritized planning. SP = spinal-cord-prioritized planning. The high-burden contrast was defined as >=2 versus 0 factors to ensure a consistent comparison across the evaluable thresholds and to avoid separation caused by all target-prioritized cases in the 3-factor group at the <=1.5 cm threshold.

**Interpretation.** The <=1.5 cm sensitivity threshold and the <=2.0 cm main-analysis threshold showed the same direction of association. Higher cumulative burden scores were associated with a greater probability of target-prioritized planning. At the <=1.0 cm threshold, regression results were not reported because only two spinal-cord-prioritized cases remained, making model estimates unstable. These findings support the robustness of the 2-cm institutional screening criterion while confirming that it functioned as a screening flag rather than a direct indication for target-prioritized planning.
