# Supplementary material for: Target-prioritized IMRT for nasopharyngeal carcinoma with tumor proximity to the spinal cord: clinical feasibility and long-term outcomes
Source: Front Oncol. 2026 Jul 6;16:1878456. doi: 10.3389/fonc.2026.1878456 (PMC13381646; doi:10.3389/fonc.2026.1878456)
Supplement: Supplementary file 3 [file Table2.docx]

**SupplTable S2. Anatomical predictors of target-prioritized planning**

*Univariable logistic regression (Part A) and cumulative cranio-cervical burden score (Part B). The burden score is the sum of three dichotomised factors with univariable P ≤ 0.05 (occipital base, occipital condyle, and atlanto-dental interval), each scored as absent (0) or present (1). Pink shading indicates univariable P < 0.05; light orange shading indicates 0.05 ≤ P < 0.10.*

**Part A. Univariable logistic regression of 25 candidate anatomical structures**

| **Anatomical structure** | **OR** | **95% CI** | **P value** |
| --- | --- | --- | --- |
| **Occipital base** | **2.728** | 1.30–5.72 | **0.008** |
| **Occipital condyle** | **2.248** | 1.29–3.91 | **0.004** |
| Hypoglossal canal | 1.610 | 0.91–2.84 | 0.099 |
| Pterygoid plate | 1.135 | 0.63–2.04 | 0.672 |
| **Atlanto-dental interval** | **3.416** | 1.00–11.65 | **0.050** |
| Carotid sheath area | 0.852 | 0.47–1.56 | 0.604 |
| Prevertebral muscles | 1.669 | 0.96–2.91 | 0.071 |
| RPLN | 1.214 | 0.50–2.94 | 0.667 |
| Petrous apex | 1.434 | 0.77–2.68 | 0.259 |
| Pharyngobasilar fascia | 1.338 | 0.67–2.66 | 0.407 |
| Cervical vertebrae | 1.160 | 0.53–2.55 | 0.711 |
| Bilateral RPLN | 0.986 | 0.45–2.15 | 0.972 |
| CLN | 1.023 | 0.37–2.83 | 0.966 |
| Intracranial involvement | 1.107 | 0.11–10.97 | 0.931 |
| Jugular foramen | 1.009 | 0.55–1.84 | 0.976 |
| Clivus | 1.751 | 0.47–6.46 | 0.400 |
| Capsular invasion | 1.621 | 0.71–3.68 | 0.249 |
| MAD of CLN >3 cm | 1.884 | 0.66–5.36 | 0.235 |
| MAD of RPLN >2 cm | 0.813 | 0.31–2.16 | 0.677 |
| Bilateral CLN metastasis | 1.087 | 0.52–2.26 | 0.824 |
| Lymph node necrosis | 0.973 | 0.44–2.16 | 0.946 |
| MAD of CLN >2 cm | 0.839 | 0.40–1.75 | 0.639 |
| Retroclival meninges | 0.809 | 0.38–1.70 | 0.577 |
| MAD of RPLN >3 cm | 0.731 | 0.06–8.29 | 0.800 |
| Spinal meninges | 0.252 | 0.05–1.28 | 0.097 |

**Part B. Cumulative cranio-cervical burden score**

| **Burden score** | **n / total (%)** | **OR (95% CI)** | **P value** |
| --- | --- | --- | --- |
| 0 factors | 29/50 (58.0%) | 1.00 (reference) | — |
| 1 factor | 23/32 (71.9%) | 1.85 (0.71–4.83) | 0.207 |
| 2 factors | 31/38 (81.6%) | 3.20 (1.18–8.71) | **0.022** |
| **3 factors** | **23/25 (92.0%)** | **8.33 (1.77–39.24)** | **0.007** |
| **Trend (per factor)** | — | **1.91 (1.30–2.80)** | **0.001** |

*OR = odds ratio. 95% CI = 95% confidence interval. The trend OR represents the increase in odds of requiring target-prioritized planning per additional factor present.*
