# Supplementary material for: Target-prioritized IMRT for nasopharyngeal carcinoma with tumor proximity to the spinal cord: clinical feasibility and long-term outcomes
Source: Front Oncol. 2026 Jul 6;16:1878456. doi: 10.3389/fonc.2026.1878456 (PMC13381646; doi:10.3389/fonc.2026.1878456)
Supplement: Supplementary file 4 [file Table3.docx]

**Supplementary Table S3. EQD2-equivalent doses for spinal cord and PRV 3-mm dosimetric parameters (target-prioritized cohort, n = 106)**

*EQD2 = D × (d + α/β) / (2 + α/β), where d = dose per fraction and α/β = 2 Gy (late spinal cord effects). Physical doses from Plan-Sum DVHs (cumulative initial + adapted plan). Physical doses are retained in the main manuscript as current spinal cord tolerance guidelines are expressed in physical dose terms.*

| **Dosimetric parameter** | **True cord Physical dose median (IQR) Gy** | **True cord EQD2**  **median (IQR) Gy** | **PRV 3-mm Physical dose median (IQR) Gy** | **PRV 3-mm EQD2**  **median (IQR) Gy** |
| --- | --- | --- | --- | --- |
| **Dmax** | 46.1 (41.6–50.9) | 39.5 (34.1–44.7) | 56.2 (53.4–59.3) | 52.8 (48.6–56.2) |
| **D0.03cc** | 44.0 (40.4–47.5) | 36.9 (32.7–41.0) | 52.2 (49.6–56.5) | 47.3 (43.6–52.8) |
| **D1cc** | 39.1 (36.7–42.9) | 31.1 (28.6–35.6) | 44.0 (41.7–47.1) | 36.8 (33.9–40.8) |
| *Dose per fraction: median 32 fractions (range 31–35). EQD2 calculated per patient using individual fraction number; values represent median EQD2 across the cohort.* | | | | |

*Note: Median true cord EQD2 Dmax = 39.5 Gy (IQR 34.1–44.7) is well below the commonly cited EQD2 tolerance range of 54–60 Gy for radiation myelopathy (α/β = 2 Gy; Kirkpatrick et al., IJROBP 2010). Median PRV 3-mm EQD2 Dmax = 52.8 Gy (IQR 48.6–56.2). These conversions are provided for cross-study comparison only.*
